# Suicidal Ideation Is Associated With Excessive Smartphone Use Among Chinese College Students

**DOI:** 10.3389/fpubh.2021.809463

**Published:** 2022-01-27

**Authors:** Qiuping Huang, Shuhong Lin, Ying Li, Shucai Huang, Zhenjiang Liao, Xinxin Chen, Tianli Shao, Yifan Li, Yi Cai, Jing Qi, Hongxian Shen

**Affiliations:** ^1^Department of Psychiatry, National Clinical Research Center for Mental Disorders, The Second Xiangya Hospital of Central South University, Changsha, China; ^2^Hunan Key Laboratory of Psychiatry and Mental Health, Hunan Medical Center for Mental Health, Chinese National Technology Institute on Mental Disorders, Institute of Mental Health of Central South University, Changsha, China; ^3^Changsha Health Vocational College, Changsha, China; ^4^Department of Psychiatry, The Fourth People's Hospital of Wuhu, Wuhu, China; ^5^Department of Psychiatry, Comorbid Somatic Diseases, Kangning Hospital of Shenzhen, Shenzhen, China; ^6^Department of Clinical Psychology, Brain Hospital of Hunan Province, Changsha, China

**Keywords:** suicidal ideation, smartphone use, mobile phone addiction, college students, social support

## Abstract

**Background:**

Suicidal ideation is the first step and a strong predictor of suicide. College students are at a considerably high risk of suicidal ideation, and smartphones are commonly used in this group. However, the relationship between suicidal ideation and smartphone use among Chinese college students is unclear. The current study aimed to investigate the prevalence of suicidal ideation among Chinese college students and its association with smartphone use and addiction factors.

**Methods:**

A total of 439 college students participated the survey. We collected the demographic information, physical health, psychosocial factors (depressive symptoms, social support, sleep quality), characteristics of smartphone use, and mobile phone addiction (MPA). Suicidal ideation was measured with a single question, “did you feel that life was not worth living in the past 1 year?”

**Results:**

The prevalence of suicidal ideation (“Yes” response) in the past year among Chinese college students was 7.5%. In binary logistic regression analysis, suicidal ideation was significantly correlated with less subjective social support (OR: 2.49, *p* = 0.049), lower utilization of social support (OR: 13.28, *p* = 0.012), more depressive symptoms (OR:4.96, *p* = 0.005), and more than 5 h of daily smartphone use (OR: 2.60, *p* = 0.025).

**Conclusion:**

Considering the widely use of smartphones in Chinese colleges and the correlation with suicidal ideation, excessive phone use among college students should be given more attention by administrators and health workers. It is necessary to obtain more information about the intention of smartphone use, make full use of smartphones for health education, and monitor excessive use of smartphones, while improving social support and coping mechanisms for depression, to identify suicidal ideation and prevent suicidal behavior among Chinese college students.

## Introduction

Suicide is a significant public health issue worldwide (1). Defined as thinking about, considering, or planning suicide, suicidal ideation is the first step and a strong predictor of suicide (2). College students are at a considerably high risk of suicidal ideation (3–8). Although many previous studies have shown the prevalence of suicidal ideation among college students worldwide, the results vary significantly as the participants reported suicidal ideation for different prevalence periods. For example, the 2-week prevalence of suicidal ideation in Korean college was 9.8% (6), that of the past 4 weeks was reported by 2% among university students (5); 6–12% for the past year (8–11); 12% for 4 years (4); and 11.4–35.3% for lifetime (7, 9, 12). According to a meta-analysis, 10.72% (ranging from 1.24 to 26%) of Chinese college students experience suicidal ideation (13), regardless of the prevalence period. It is necessary to explore the incidence of suicidal ideation among Chinese college students over the past 12 months.

Diverse factors have been reported to be related with suicidal ideation among college students, such as external environment (e.g., living alone, economic class, religious practice, peer problems, suicide attempts in the family or among friends), mental health (e.g., depressive symptoms, sexual orientation, probable obsessive-compulsive disorder, hopelessness, poor social support, maladaptation), and behaviors (e.g., previous suicide attempts, poor academic performance, alcohol consumption, and pathological Internet use) (10, 14–17). Evidence from empirical studies about college students and adolescents has shown that there is a relationship between suicidal ideation and addiction, including substance abuse or dependence (18), alcohol or tobacco consumption (19), pathological Internet use or addiction (15), as well as mobile phone use (20, 21).

Smartphone has been an essential tool in our daily life. It is even applied as an intervention approach to cope with suicidal crises based on its functions (e.g., text messaging, call, Apps) (22–24). However, it should not be ignored that excessive smartphone use or addiction has become a global public health problem, particularly among college students. Previous studies have shown the relationship between suicidal ideation and mobile phone use or dependence in adolescents; for example, both early and late adolescents who use mobile phones past their bedtime may have more suicidal feelings (21). For adolescents with problematic phone use, a good family function is a protective factor for reducing the risk of suicidal ideation (20). Suicidal tendencies may be one of the risky behaviors associated with cellular phone use (19). Research involving Chinese vocational school students found that mobile phone dependence was positively correlated with suicidal ideation, and the risk of suicide was much higher among students with mobile phone dependence and depressive symptoms (25).

To date, most studies exploring suicidal ideation and mobile phone use have focused on adolescents (21). Research on suicidal ideation in college students has focused more on its relationship with Internet use/addiction (26), another critical problem for this group. However, data on phone use are more convenient and readily available than those on Internet use. Remarkably little is known about their relationship between mobile phone addiction (MPA) and suicidal ideation among Chinese college students. Furthermore, the suicidal ideation rate and its association with smartphone use may differ from different cultures (9), and it is meaningful to examine its prevalence and relationship among Chinese college students.

The aim of the current study was to investigate the prevalence of suicidal ideation among Chinese college students and determine its association with smartphone use characteristics and MPA factors.

## Materials and Methods

### Subjects

We conducted a cross-sectional survey in May 2016 at Changsha Health Vocational College. All internal students from the college's Department of Pharmacy were recruited through cluster sampling. We excluded students unwilling to participate in the survey or those with severe physical and mental illnesses.

The study protocol was approved by the Institutional Review Board of the Changsha Health Vocational College. All students agreed to participate in the study and signed informed consent.

### Procedures

After signing written consent, all participants were invited to complete the self-report questionnaires in their classrooms. Two professional investigators distributed the questionnaires and instructed the participants to complete them.

### Measures

#### Social-Demographic Characteristics

A detailed self-administered questionnaire was used to collect general information on sociodemographic characteristics, including age, gender, level of family income (poor, fair, and good), and place of origin (rural vs. urban).

#### Psychosocial Factors

Psychosocial factors include depressive symptoms, social support, and sleep quality. Depressive Symptoms, adapted from the University Personality Inventory (UPI), are measured on the scale of depressive symptoms (SDS) which comprises 12 items, including suicidal thoughts, poor appetite, pessimism, distraction, restlessness, no interest in anything, negative mood, lack of confidence, lack of judgment, feeling self-abased, physically exhausted, frequent insomnia, and hesitation. The score of the item, “having suicidal thoughts” on the UPI, was not included in the total score of the SDS to avoid an overlap between suicidal thoughts due to depressive symptoms and the main outcome of this study. Participants responded “yes” or “no” to each item and given scores or 1 or 0, respectively. The total score ranges from 0 to 12, and higher scores represent more severe depressive symptoms. The Cronbach's α coefficient of SDS in this sample of college students was 0.751 (27).

Two subscales of the validated Chinese Social Support Rating Scale were adopted to assess the utilization of social support and subjective social support, respectively (28, 29). Higher scores on each subscale indicate higher levels of social support.

Sleep Quality was evaluated using the Pittsburgh Sleep Quality Index (PSQI) (30). The validated Chinese PSQI, with good reliability and validity (31), consists of 18 items. The total score ranged from 0 to 21. Higher scores indicate poorer sleep quality; the cut-off score in China for poor sleep quality is usually ≥8 (32).

#### Physical Health

Physical health included self-reported good health and incidence of headaches (27). Self-reported good health was measured using one question: “During the past year, did you often feel that you were in good physical health?” Respondents with the answer “yes” indicated having good physical health. Incidence of headaches was measured using the question, “During the past year, did you often suffer from headaches?” As before, respondents who selected “yes” were considered to have suffered from headaches.

#### Smartphone Use and MPA

Smartphone use characteristics included time spent on daily smartphone use and mobile internet use (hours), smartphone charge per month (yuan), length of smartphone use (years), and MPA. In addition, the mobile phone addiction index (MPAI) was used to assess MPA (33). The MPAI consists of 17 items and is scored on a 5-point Likert scale from 1 = never to 5 = always. The MPAI includes four dimensions of MPA: inability to control carving, withdrawal, or escape, feeling anxious and lost, and productivity loss. Higher scores reveal greater severity of MPA.

#### Suicidal Ideation

Suicidal ideation was measured with a single question: “Did you feel that life was not worth living in the past 1 year?” The response options were yes or no. If the answer was “Yes,” the student was considered as experiencing suicidal ideation in the past year. Similar methodology has been followed in previous studies on suicidal ideation (13, 21, 34, 35).

### Statistical Analysis

All the statistical analyses were conducted by SPSS software. The prevalence of suicidal ideation was also determined. Differences between participants with and without suicidal ideation in terms of each variable were compared using the chi-square (χ^2^) test and Fisher's exact test. All significant factors in the univariate analysis were entered in Binary logistic regression, which was used to recognize factors associated with suicidal ideation. Odds ratios (ORs) and 95% confidence intervals (CIs) were calculated for each variable. The statistical significance level was set at *p* < 0.05.

## Results

After excluding four students who submitted incomplete forms, a total of 439 completed questionnaires were obtained. The average age of the participants was 18.8 years (SD: 1.7, range: 15–24). Generally, there are more females than males in health vocational colleges; thus, 381(86.8%) students in this study were female. The average duration of owning a smartphone was 3.9 years (SD: 2.3). Detailed demographic and psychological factors, characteristics of smartphone use, and MPA are shown in Table 1.

**Table 1:** Socio-demographic and smartphone use characteristics of college students and prevalence rates of suicidal ideation by variables.

**Variables**		**No**.	**Suicidal ideation (%)**		**χ^2^**	***p***
			**Yes**	**No**		
			**(*n* = 33)**	**(*n* = 406)**		
Gender	Male	58	4 (6.9)	54 (93.1)		
	Female	381	29 (7.6)	352 (92.4)	0.037^#^	0.847
Age	≤ 19 years	274	25 (9.1)	249 (90.9)		
	≥20 years	165	8 (4.8)	157 (95.2)	2.708	0.100
Place of origin	Rural	246	18 (7.3)	228 (92.7)		
	Urban	193	15 (7.8)	178 (92.2)	0.032	0.858
Self-rated family economic status	Good	26	1 (3.8)	25 (96.2)		
	Fair	256	17 (6.6)	239 (93.4)		
	Poor	157	15 (9.6)	142 (90.4)	1.724^#^	0.422
Subjective social support^*^	≤ 9	227	26 (11.5)	201 (88.5)		
	>9	212	7 (3.3)	205 (96.7)	10.479	0.001
Utilization of social support^*^	≤ 8	288	32 (11.1)	256 (88.9)		
	>8	151	1 (0.7)	150 (99.3)	15.557	<0.001
Depressive symptoms^*^	≤ 2	210	4 (1.9)	206 (98.1)		
	>2	229	29 (12.7)	200 (87.3)	18.24	<0.001
Good physical health	Yes	381	24 (6.3)	357 (93.7)		
	No	58	9 (15.5)	49 (84.5)	6.153^#^	0.027
Headache	Yes	92	15 (16.3)	77 (83.7)		
	No	347	18 (5.2)	329 (94.8)	12.928	<0.001
**Characteristics of phone use**
Years of smartphone use^*^	≤ 4	267	24 (9.0)	243 (91)		
	>4	172	9 (5.2)	163 (94.8)	2.123	0.145
Monthly smartphone charge (RMB) ^*^	≤ 50	236	17 (7.2)	219 (92.8)		
	>50	203	16 (7.9)	187 (92.1)	0.072	0.788
Hours of daily smartphone use^*^	≤ 5	249	10 (4.0)	239 (96)		
	>5	190	23 (12.1)	167 (87.9)	10.144	0.001
Hours of mobile internet use^*^	≤ 5	260	15 (5.8)	245 (94.2)		
	>5	179	18 (10.1)	161 (89.9)	2.802	0.094
**Mobile phone addiction index**
Inability to control carving^*^	≤ 14	239	12 (5.0)	227 (95)		
	>14	200	21 (10.5)	179 (89.5)	4.702	0.030
Feeling anxious and lost^*^	≤ 7	258	18 (7.0)	240 (93)		
	>7	181	15 (8.3)	166 (91.7)	0.263	0.608
Withdrawal or escape^*^	≤ 7	259	18 (6.9)	241 (93.1)		
	>7	180	15 (8.3)	165 (91.7)	0.292	0.589
Productivity loss^*^	≤ 5	231	13 (5.6)	218 (94.4)		
	>5	208	20 (9.6)	188 (90.4)	2.503	0.114
Poor sleep quality	Yes	43	6 (14.0)	37 (86)		
	No	396	27 (6.8)	369 (93.2)	2.841^#^	0.119

* *All continuous variables were dichotomized at the median value*.

# *Fisher's exact test*.

A total of 33 students (7.5%) had suicidal ideation (“Yes” response). The chi-square (χ^2^) tests (see Table 1) showed that students with suicidal ideation were more likely to have less subjective social support (*p* < 0.001), lower utilization of social support (*p* < 0.001), more depressive symptoms (as indicated by a score of three or more) (*p* < 0.001), lack good physical health (*p* = 0.027), and suffer from headaches (*p* < 0.001). Students with suicidal ideation were also found to use a smartphone for more than 5 h a day (*p* < 0.001), and have more serious MPA in terms of inability to control craving (*p* = 0.030).

The binary logistic regression analysis (see Table 2) showed that suicidal ideation among college students was significantly associated with less subjective social support (OR:2.49, *p* = 0.049), lower utilization of social support (OR:13.28, *p* = 0.012), more depressive symptoms (OR:4.96, *p* = 0.005), and more than 5 h of daily smartphone use (OR:2.60, *p* = 0.025).

**Table 2:** Binary logistic regression of factors associated with suicidal ideation.

**Variable**	**Risk level**	**Reference level**	**Unstandardized coefficient**	**Standard error**	**Wald **χ^2^****	***p***	**OR (95%CI)**
Subjective social support^*^	≤ 9	>9	0.910	0.462	3.881	0.049	2.49 (1.01, 6.15)
Utilization of social support^*^	≤ 8	>8	2.587	1.033	6.273	0.012	13.28 (1.76, 100.55)
Depressive symptoms^*^	>3	≤ 3	1.602	0.568	7.940	0.005	4.96 (1.63, 15.12)
Hours of daily smartphone use^*^	>5	≤ 5	0.956	0.427	5.009	0.025	2.60 (1.13, 6.01)

* *All continuous variables were dichotomized at the median value*.

## Discussion

The current study investigated the prevalence of suicidal ideation among Chinese college students and its association with smartphone use and MPA. We found a 7.5% prevalence of suicidal ideation, significantly related to less subjective social support, lower utilization of social support, more depressive symptoms, and more than 5 h of daily smartphone use in Chinese college students.

### Prevalence of Suicidal Ideation

Our study showed that the prevalence of suicidal ideation among Chinese college students was 7.5%, slightly lower than the overall pooled prevalence of 10.7% from a meta-analysis of 41 studies involving 60,339 Chinese college students (13). The result is quite similar to other research on suicidal ideation among university students in other countries during the same prevalence period (12 months), such as 7.2% among Brazilian medical students (10) and 6% in the USA (11) but lower than the 10.7% in Portugal (8) and 11.3 and 12% in Austria and Turkey (9). The difference in prevalence between countries may be due to the different instruments used to assess suicidal ideation or due to cultural differences (9, 36). The prevalence finding indicates that three in every 40 Chinese college students reported the presence of suicidal ideation in the past year, which should put education administrators on alert.

### Suicidal Ideation and Mental, Physical Health

Suicidal ideation was significantly related to socio-psychological factors (depressive symptoms and lack of social support) and poor physical health, consistent with previous studies (14, 20, 37). In the current study, 29 (87.9%) reported depressive symptoms among 33 participants with suicidal ideation. Depression is strongly associated with suicidal ideation among college students, consistent with studies in the USA (38) and Brazil (14, 37). For example, among the 267 Brazilian students who had depressive symptoms, 21.4% had thought of taking their life in the previous 30 days. People suffering from depression may have negative thoughts about life, leading to suicidal ideation and even suicide attempts. On the contrary, the continuous presence of suicidal ideation may increase the severity of depressive symptoms. In the binary logistic regression analysis, a strong association between suicidal ideation and depressive symptoms remained.

Social support was also strongly related to suicidal ideation among college students, similar to previous studies (13). College students with less subjective social support and lower utilization of social support may experience higher levels of suicidal ideation given that social support is an important protective factor against suicidal ideation. Conversely, high levels of family cohesion and family support were significantly correlated with lower levels of suicidal ideation (39). For college students, support mainly comes from family, friends, and school. Therefore, it is essential to evaluate the support system, improve subjective social support, and make full use of it.

In this study, we found a positive correlation between suicidal ideation and poor physical health. College students who thought they did not have good physical health or had headaches in the past year may be at greater risk of suicidal ideation. Several studies have confirmed that individuals suffering from diseases or pain may be more likely to end their lives (17). Low physical activity was related with suicidal ideation among Chinese college students (40). College students should take enough physical training and keep physical health. Meanwhile, teachers should pay more attention to those students with poor physical health.

### Suicidal Ideation and Smartphone Use and MPA

The current study revealed that students with suicidal ideation were more likely to use phones for more than 5 h a day and have more serious MPA in terms of inability to control craving. The binary logistic regression analysis found that only the relationship between suicidal ideation and excessive smartphone use is significant. This finding is consistent with prior studies on adolescents. Oshima et al. (21) found that suicidal feelings were significantly correlated with nocturnal mobile phone use among Japanese adolescents. A 1-year-follow-up study among Chinese adolescents revealed that excessive use of mobile phone was an important risk factor of self-harm (41), which is also an early predictor of suicidal behaviors like suicidal ideation. However, inconsistent with other studies, we did not find a strong relationship between suicidal ideation and MPA. A previous study highlighted the connection between problematic cellular phone use and suicidal ideation among Chinese adolescents (20), in which problematic cellular phone use included the symptoms and participants' subjective functional impairment in the preceding year. The reasons for the inconsistent results may be due to the different tools of measurements used. Furthermore, when assessing the contribution to suicidal ideation among college students simultaneously, excessive smartphone use contributes more than MPA. Further studies are required to examine these relationships.

However, the exact mechanism underlying the relationship between suicidal ideation and smartphone use remains unknown. Smartphone use can either have a devastating effect, or a protective effect on those with suicidal ideation. On one hand, excessive smartphone use may affect suicidal ideation through psychological pathways, such as depression, poor sleep quality, chaotic lifestyle, low self-esteem, and low-income family function (20). For example, studies suggested family function moderately affected the association between problematic phone use and suicidal ideation and good family function may help to decrease the risks of suicidal ideation (20). Depression and interpersonal problems mediated the association between high-intensity phone use and suicide ideation (42). On the other hand, when students intend to harm themselves or even end their lives, they may benefit from using mobile phones (19). In some studies on Internet addiction (43), smartphone use is viewed either as a threat or an opportunity. Our study agrees that college students with suicidal ideation may use mobile phones more frequently, but not have MPA. However, individuals' frequent smartphone use may be due to two factors: getting helps through interventions (e.g., “online counseling,” support group, self-evaluation), and getting information about suicide (using the phone as a medium to learn methods to end their lives) (22, 44). Smartphone use may be a cope style for those individuals with suicidal thoughts. Therefore, further studies are needed to explore the actual reasons for the association between smartphone use and suicidal ideation.

The following limitations of this study need to be acknowledged. First, this was a cross-sectional study; thus, the causal relationship could not be clarified when significant associations with excessive smartphone use, depressive symptoms, and social support were observed. Therefore, longitudinal follow-up studies are essential. Second, we used a self-report questionnaire, which can result in a recall bias. Third, our sample was recruited from a health vocational college, which limits the representativeness of the sample. Therefore, caution is necessary when generalizing the findings to other samples. More large-scale and prospective studies with well representative should be conduct to confirm these associations.

## Conclusions

In summary, approximately three out of 40 Chinese college students experienced significant suicidal ideation in the past year, and suicidal ideation was significantly associated with poor social support, depression, and excessive smartphone use. Considering the widely use of smartphones in Chinese colleges and its relationship with suicidal ideation, administrators and health workers ought to give greater attention and concern to the excessive phone use among college students. Therefore, it is necessary to obtain more information about the intention of smartphone use, make full use of smartphones for health education and monitor excessive use of smartphones, while improving social support and coping mechanisms for depression, to help identify suicidal ideation and prevent suicidal behavior among Chinese college students.

## Data Availability Statement

The original contributions presented in the study are included in the article/supplementary material, further inquiries can be directed to the corresponding author/s.

## Ethics Statement

The studies involving human participants were reviewed and approved by the Institutional Review Board of the Changsha Health Vocational College. The patients/participants provided their written informed consent to participate in this study.

## Author Contributions

QH, SL, JQ, and HS conceived and designed the study. YinL, SH, ZL, XC, TS, and YifL collected and analyzed the data. QH and SL wrote the manuscript. YC, JQ, and HS revised the manuscript. All authors approved the final manuscript to be published.

## Funding

This work was supported by National Natural Science Foundation of China (No. 81971249), National Key R&D Program of China (No. 2020YFC2005300), and Natural Science Foundation of Hunan Province (No. 2020JJ4782).

## Conflict of Interest

The authors declare that the research was conducted in the absence of any commercial or financial relationships that could be construed as a potential conflict of interest.

## Publisher's Note

All claims expressed in this article are solely those of the authors and do not necessarily represent those of their affiliated organizations, or those of the publisher, the editors and the reviewers. Any product that may be evaluated in this article, or claim that may be made by its manufacturer, is not guaranteed or endorsed by the publisher.
